# Directed elimination of senescent cells by inhibition of BCL-W and BCL-XL

**DOI:** 10.1038/ncomms11190

**Published:** 2016-04-06

**Authors:** Reut Yosef, Noam Pilpel, Ronit Tokarsky-Amiel, Anat Biran, Yossi Ovadya, Snir Cohen, Ezra Vadai, Liat Dassa, Elisheva Shahar, Reba Condiotti, Ittai Ben-Porath, Valery Krizhanovsky

**Affiliations:** 1 Department of Molecular Cell Biology, The Weizmann Institute of Science, Rehovot 76100, Israel; 2 Department of Developmental Biology and Cancer Research, Institute for Medical Research Israel-Canada, The Hebrew University-Hadassah Medical School, Jerusalem 91120, Israel

## Abstract

Senescent cells, formed in response to physiological and oncogenic stresses, facilitate protection from tumourigenesis and aid in tissue repair. However, accumulation of such cells in tissues contributes to age-related pathologies. Resistance of senescent cells to apoptotic stimuli may contribute to their accumulation, yet the molecular mechanisms allowing their prolonged viability are poorly characterized. Here we show that senescent cells upregulate the anti-apoptotic proteins BCL-W and BCL-XL. Joint inhibition of BCL-W and BCL-XL by siRNAs or the small-molecule ABT-737 specifically induces apoptosis in senescent cells. Notably, treatment of mice with ABT-737 efficiently eliminates senescent cells induced by DNA damage in the lungs as well as senescent cells formed in the epidermis by activation of p53 through transgenic p14^ARF^. Elimination of senescent cells from the epidermis leads to an increase in hair-follicle stem cell proliferation. The finding that senescent cells can be eliminated pharmacologically paves the way to new strategies for the treatment of age-related pathologies.

## Abstract (web summary)

The accumulation of senescent cells within tissues plays a role in numerous age-related pathologies. Yosef and Pilpel *et al*. demonstrate that the resistance of these cells to apoptosis is driven by upregulation of survival proteins, whose pharmacological inhibition triggers senescent cell elimination in mice.

## Graphical abstract

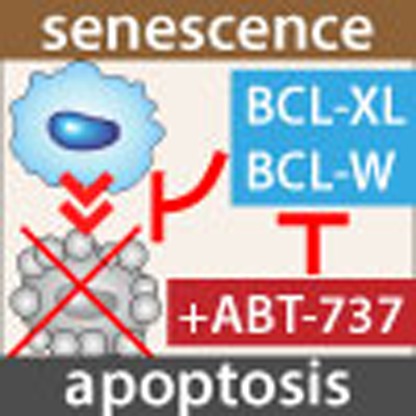

Cellular senescence is a stable form of cell cycle arrest that limits the proliferative potential of cells. Senescence is triggered in many cell types in response to diverse forms of cellular stress^1^,^2^,^3^,^4^. Activation of senescence in premalignant lesions acts as a potent barrier to tumourigenesis. In addition, senescence has been shown to contribute to the cytotoxicity of anti-cancer agents and to support tissue repair by limiting excessive proliferation of cells^5^,^6^,^7^,^8^,^9^,^10^.

While short-term induction of cellular senescence can be beneficial in various settings, long-term retention of senescent cells appears to be deleterious to the organism. These cells commonly secrete pro-inflammatory factors that can facilitate their removal by the immune system in some settings^11^. However, if senescent cells are retained in tissues, these factors can promote local inflammation, tissue aging, tissue destruction and, potentially, tumourigenesis and metastasis in a cell non-autonomous manner^1^,^3^,^12^,^13^. The elimination of senescent cells in a mouse model of premature aging was shown to reduce tissue aging^14^. Understanding how senescent cell viability is regulated at the molecular level could therefore point to pharmacological targets allowing specific elimination of senescent cells *in vivo.* Such elimination would allow the assessment of the functional importance of cellular senescence in different pathological conditions, and, potentially, lead to development of therapies.

Senescent cells have been reported to be resistant to extrinsic and intrinsic pro-apoptotic stimuli^15^,^16^,^17^. While the mechanisms driving senescence are well studied, understanding of the mechanisms endowing these cells with increased survival capacity is limited. The BCL-2 protein family plays a central role in cell death regulation by diverse mechanisms, including apoptosis and autophagy^16^,^18^,^19^. This family includes the anti-apoptotic proteins BCL-2, BCL-W, BCL-XL, MCL-1 and A1, and is intensively studied as a target for pharmacological intervention in cancer^20^,^21^. We set out to evaluate the individual contributions of each of these BCL-2 family members and their combinations to the viability of senescent cells. We found that the increased presence of BCL-W and BCL-XL underlies senescent cell resistance to apoptosis, and that their combined inhibition leads to senescent cell death. We show that a small-molecule inhibitor targeting the BCL-2, BCL-W and BCL-XL proteins (ABT-737) causes preferential apoptosis of senescent cells, both *in vitro* and *in vivo*, and eliminates these cells from tissues, opening the door for targeted elimination of senescent cells.

## Results

### Senescent cells upregulate proteins of the Bcl-2 family

**Figure 1:**
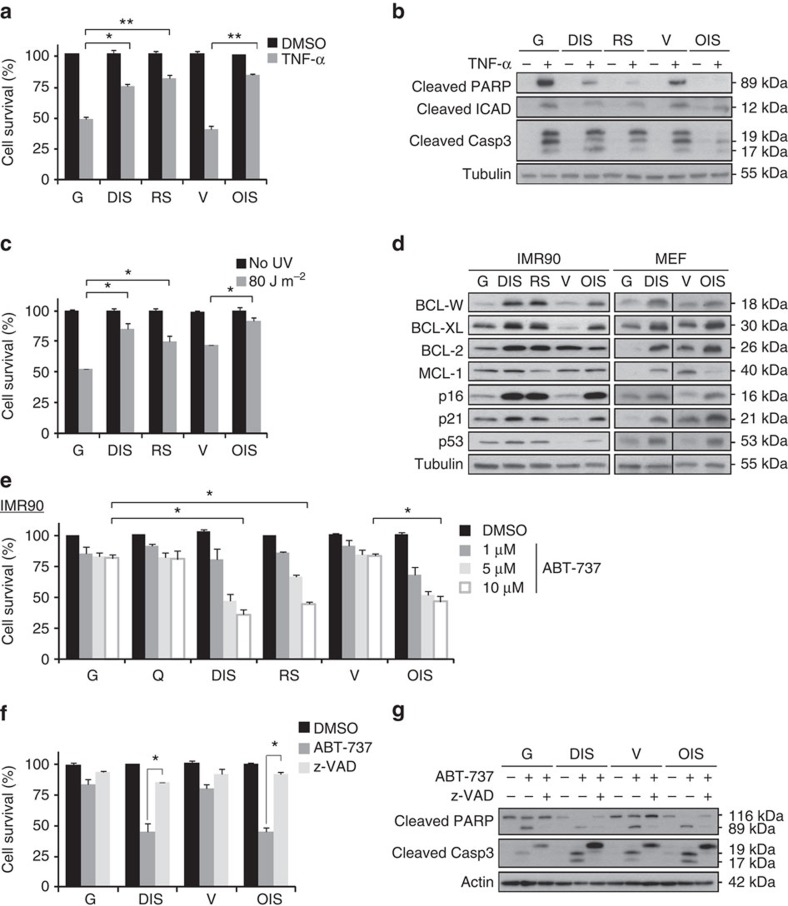
BCL-2 family members are elevated in senescent cells and provide resistance to apoptosis. (**a**) IMR-90 human fibroblasts that were induced to senesce either through DNA damage (DIS), replicative exhaustion (RS) or oncogene expression (OIS), as well as controls proliferating cells (growing, G) and empty vector-transfected (V) cells, were treated for 10 h with TNF-α and CHX (TNF-α) or with vehicle (DMSO). Cell survival relative to vehicle-treated cells was determined by quantification of the remaining adherent cells. Histograms indicate the percentages of surviving senescent (DIS, RS and OIS) cells compared with G or V controls. Data are presented as mean±s.e.m of three repeats, performed in triplicates. (**b**) Western blot analysis of cleaved PARP, ICAD and caspase-3 proteins from senescent and control IMR-90 cells (labelled as in **a**), indicating levels of apoptosis after treatment with TNF-α and CHX. (**c**) Percentage survival of senescent and control cells (labelled as in **a**) 24 h after UV irradiation (80 J m^−2^). Data are presented as mean±s.e.m of three repeats, performed in triplicates. (**d**) Expression of BCL-2 family members (BCL-W, BCL-XL, BCL-2 and MCL-1) and of senescence effector proteins (p16, p21, p53) in senescent (DIS, RS, and OIS) and control (G, V) cells of both human (IMR-90) and mouse (MEF) origin. (**e**) Percentage survival of senescent and control cells (as in **a**) as well as in quiescent (Q) control cells after treatment for 24 h with the indicated concentrations of ABT-737, an inhibitor of BCL-W, BCL-XL and BCL-2. Data are presented as mean±s.e.m of three repeats, performed in triplicates. (**f**) Percentage survival of senescent (DIS and OIS) and control (G and V) cells treated with ABT-737 (10 μM) for 24 h with or without pretreatment for 6 h with the pan-caspase inhibitor z-VAD-fmk. Data are presented as mean±s.e.m of three repeats, performed in triplicates. (**g**) Western blot analysis of cleaved PARP and caspase-3 in the samples described in **f**. Data in **b**,**d**,**g** are representative blots of at least two independent experiments each. Data were analysed using Student's *t*-test. *P<0.05, **P<0.005.

To examine the resistance of senescent cells to intrinsic and extrinsic apoptotic pathways, we induced senescence in normal primary human fibroblasts (IMR-90), one of the most widely used cell models for the study of this program^5^,^22^,^23^. This was done by three different means: treatment with the DNA-damaging agent etoposide to induce DNA-damage-induced senescence (DIS); replicative exhaustion for replicative senescence (RS); and expression of oncogenic *H-Ras*^*V12*^ for oncogene-induced senescence (OIS). These cells were compared with proliferating (growing) vehicle-treated cells or empty vector-transduced cells. Senescent and control IMR-90 cells were then treated with tumour necrosis factor-α (TNF-α) and cycloheximide (CHX) together, or with UV irradiation, to induce extrinsic or intrinsic apoptotic pathways, respectively. Following TNF-α treatment, the survival of senescent cells was significantly higher than that of control cells (76 or 82% versus 49% for DIS or RS cells versus growing cells (G); 85% versus 40% for OIS cells versus vector-transduced cells (V); Fig. 1a). The lower levels of apoptosis in senescent cells were confirmed by decreased cleavage of three markers indicative of apoptosis: poly-ADP-ribose polymerase (PARP); inhibitor of caspase-activated DNase (ICAD); and caspase-3 (Fig. 1b). Similarly, senescent cells were more resistant to UV irradiation than control cells (52% versus 86% or 75% for control (G) cells versus DIS or RS cells; 72% versus 92% for control (V) cells versus OIS cells; Fig. 1c). The above findings established that senescent cells are more resistant than non-senescent cells to both intrinsic and extrinsic pro-apoptotic stimuli.

We hypothesized that an increase in the levels of anti-apoptotic proteins accounts for the resistance of senescent cells to apoptosis. Among the essential regulators of both intrinsic and extrinsic apoptosis are members of the BCL-2 protein family^18^,^19^. We measured the levels of the anti-apoptotic proteins BCL-W, BCL-XL, BCL-2 and MCL-1 (ref. 20) in senescent and control (G) cells. The expression levels of BCL-W, BCL-XL and BCL-2 were increased in both human (IMR-90) cells and mouse embryonic fibroblasts (MEFs), in which senescence had been induced by DNA damage or *H-Ras*^*V12*^ expression (Fig. 1d). Expression of MCL-1 varied between stress-stimulus conditions (Fig. 1d).

In light of the consistent upregulation of BCL-W, BCL-XL and BCl-2 observed in all tested types of senescent cells, we examined the effects of their inhibition on cell viability using ABT-737, a potent small-molecule inhibitor of BCL-2, BCL-XL and BCL-W^24^,^25^. Human senescent cells of all three types were significantly more sensitive than control cells to treatment with ABT-737, showing up to 65% death at the highest concentration tested (Fig. 1e). The same effect was observed following ABT-737 treatment of senescent and control MEFs (Supplementary Fig. 1a).

To determine whether ABT-737-treated senescent cells underwent apoptosis, we treated DIS and OIS cells with ABT-737 in the presence of the pan-caspase inhibitor z-VAD-fmk. This inhibitor completely prevented the cytotoxic effect of ABT-737 on senescent cells (Fig. 1f), and abolished the increased cleavage of PARP and caspase-3 in the senescent cells (Fig. 1g). These findings indicate that BCL-W, BCL-XL and BCL-2 confer resistance of senescent cells to apoptosis, and their inhibition by ABT-737 triggers cell death specifically in these cells.

### BCL-W and BCL-XL maintain the viability of senescent cells

**Figure 2:**
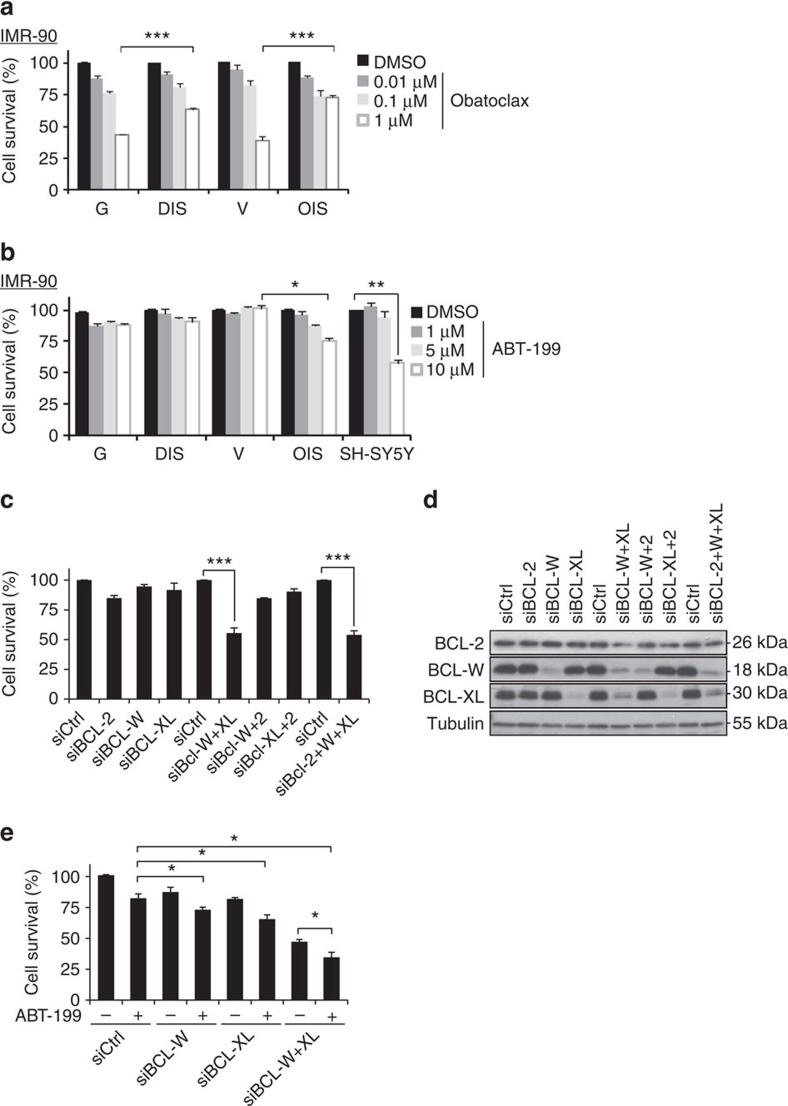
BCL-W and BCL-XL maintain the viability of senescent cells. (**a**) IMR-90 human fibroblasts (DIS, and OIS cells, as well as control G or V cells) were treated for 24 h with the indicated concentrations of Obatoclax or with vehicle (DMSO). Cell survival relative to vehicle-treated cells was determined by quantification of the remaining adherent cells. Histograms indicate the percentages of surviving senescent (DIS and OIS) cells compared with G or V controls. Data are presented as mean±s.e.m of three repeats, performed in triplicates. (**b**) Percentage survival of the cells described in **a** following 24 h treatment with the indicated concentrations of the BCL-2 inhibitor ABT-199 and of SH-SY5Y cells, which express high BCL-2 levels and serve as positive control for response to the drug. Data are presented as mean±s.e.m of three repeats, performed in triplicates. (**c**) Percentage survival of DIS cells transduced with siRNAs targeting *BCL-2*, *BCL-W* and *BCL-XL* or their combinations as indicated. (**d**) Western blot analysis of BCL-2, BCL-W and BCL-XL following siRNA treatment of DIS cells. (**e**) Percentage survival of DIS cells transduced with siRNAs targeting *BCL-W*, *BCL-XL* or both, with or without treatment with the BCL-2 inhibitor ABT-199 (10 μM). Data are presented as mean±s.e.m of three repeats, performed in triplicates. Data were analysed using Student's *t*-test. *P<0.05, **P<0.005, ***P<0.0005.

ABT-737 has a high affinity to BCL-W, BCL-XL and BCL-2, but not to MCL-1 (ref. 24). To find out whether broad inhibition of BCL-2 family members by a different inhibitor also results in increased elimination of senescent cells, we treated IMR-90 cells with obatoclax, an inhibitor of BCL-W, BCL-XL, BCL-2, and relatively selective inhibitor of MCL-1 (ref. 26). Surprisingly, obatoclax induced significantly more death of control cells than of senescent cells (44 versus 64% of remaining adherent cells for growing cells versus DIS cells; 39 versus 72% of remaining adherent cells for V cells versus OIS cells; Fig. 2a). Obatoclax, therefore, cannot induce specific death of senescent cells.

Next, we studied the individual contributions of BCL-W, BCL-XL and BCL-2 to the resistance of senescent cells to apoptosis. No specific inhibitors are available for BCL-W or BCL-XL, but BCL-2 can be specifically inhibited by ABT-199 (ref. 27). Interestingly, only OIS cells showed a significant dose-dependent reduction in cell viability in response to ABT-199 treatment (Fig. 2b). The reduction in viability of OIS cells in response to ABT-199 treatment might be attributable to lower levels of BCL-2 in these cells (Fig. 1d), to the oncogenic context that increases dependence on BCL-2, or to both. ABT-199 had no effect, however, on the viability of either senescent or growing MEFs (Supplementary Fig. 2a). These results indicated that BCL-W and BCL-XL account for most of the resistance of senescent cells to apoptosis.

To further study the contributions of BCL-W and BCL-XL to the resistance of senescent cells to apoptosis we used siRNAs to silence both genes, individually or together. siRNA mixtures directed against either *BCL-W* or *BCL-XL* efficiently reduced their protein levels, but silencing of these genes individually resulted in only minor reductions in DIS cell viability (Fig. 2c,d). In contrast, combined knockdown of BCL-W and BCL-XL had a synergistic effect, leading to a dramatic reduction (53%) in cell viability (Fig. 2c), comparable to that induced by ABT-737 treatment. We were not able to test the effect of *BCL-2* silencing, as the siRNAs against this gene caused only a minor reduction in its protein level despite an observed reduction in its mRNA level (Fig. 2d and Supplementary Fig. 2b). To further delineate the relative contributions of the three BCL-family members, we treated senescent cells with siRNAs against *BCL-W*, *BCL-XL* or their combination, as well as with the BCL-2 inhibitor ABT-199 (Fig. 2e). Similarly, inhibition of BCL-2 by ABT-199 reduced cell viability when the inhibitor was combined with siRNAs against *BCL-W* (by 14%), against *BCL-XL* (by 16%) or against both (by 13%). However, this additional effect was only additive, not synergistic as observed in the case of combined inhibition of BCL-W and BCL-XL. Thus, while all three family members contribute to the viability of DIS cells, BCL-W and BCL-XL play a more prominent role than BCL-2.

### Regulation of expression of BCL-W and BCL-XL proteins

**Figure 3:**
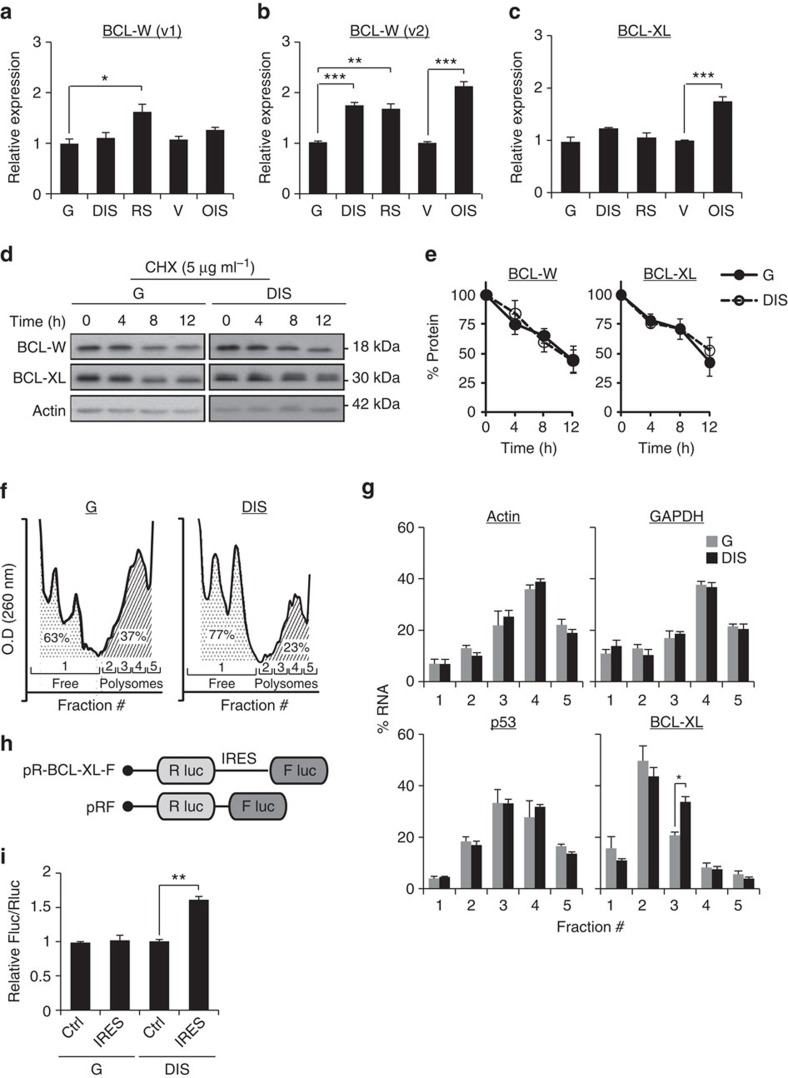
Molecular mechanisms regulating BCL-W and BCL-XL protein level in senescent cells. (**a**,**b**) mRNA expression levels of *BCL-W* variants 1 and 2 (v1 and v2, respectively) in IMR-90 human fibroblasts (DIS, RS and OIS cells) as well as IMR-90 control (G or V) cells. Data are presented as mean±s.e.m of three repeats. (**c**) mRNA expression levels of *BCL-XL* in IMR-90 human fibroblasts (DIS, RS and OIS cells) as well as IMR-90 control (G or V) cells. Data are presented as mean±s.e.m of three repeats. (**d**) Western blot analysis of BCL-W and BCL-XL following inhibition of protein translation by CHX at the indicated time points after treatment. (**e**) Quantification of protein levels in **d**. Data are presented as mean±s.e.m of three independent experiments. (**f**) Polysomal profiles of control (G) and senescent (DIS) cells after sucrose gradient fractionation. The area under the curve was calculated for monosomes (fraction #1) and polyribosomes (polysomes, fractions 2−5), and indicates a lower polysomal content in DIS cells than in G cells. Data are presented as mean±s.e.m of three independent experiments. (**g**) Distribution of the mRNAs of *GAPDH, Actin, p53* and *BCL-XL* in ribosomal fractions in senescent (DIS) cells and control (G) cells. The presented changes are relative to total mRNA. Values indicate the average percentage of mRNA in each fraction of total mRNA derived from three independent ribosomal fractionations. (**h**) Schematic representation of the bicistronic constructs. The BCL-XL construct contains the IRES of 5′-UTR *BCL-XL* (ref. 33). (**i**) Relative IRES activity is represented by the Fluc/Rluc ratio for all constructs transfected into IMR-90 cells. Data are presented as mean±s.e.m of three independent experiments. Data were analysed using Student's *t*-test. *P<0.05. **P<0.005.

We next set out to uncover the molecular basis for the accumulation of BCL-W and BCL-XL proteins in senescent cells. First, we assessed the transcript levels of these genes. The *BCL-W* gene encodes two transcript variants that give rise to the same protein and are initiated from different transcription start sites. Quantitative real-time PCR analysis revealed that *BCL-W* variant-1 (v1) mRNA level was elevated only in RS cells relative to controls (G) (Fig. 3a). In contrast, a significant elevation in *BCL-W* variant-2 (v2) mRNA level was detected in all senescent cells tested relative to controls (Fig. 3b). We therefore concluded that the increase in transcription of BCL-W protein may account for the observed elevation in its protein level in senescent cells. *BCL-XL* mRNA levels were elevated only in OIS cells relative to control cells (V) (Fig. 3c). Therefore, transcriptional upregulation of this gene might be responsible for its elevation in OIS cells but cannot account for the observed increase in its protein expression in DIS or RS cells.

The accumulation of BCL-XL protein in DIS cells could result from differences in its translation or degradation rates. First, we set to examine whether alterations in protein degradation account for the increased levels of BCL-XL protein in DIS cells. We treated cells with the protein synthesis inhibitor CHX, and monitored the rate of protein degradation over time. After 12 h of treatment the protein levels of both BCL-XL and BCL-W were reduced by half (Fig. 3d and Supplementary Fig. 3a) and there was no difference in the protein degradation rates between control and DIS cells (Fig. 3e). Likewise, we observed no difference in protein levels following inhibition of the proteasome machinery; this is in contrast to the dramatic increase observed in levels of TP53 (p53) protein, which is known to be regulated by the proteasome^28^ (Supplementary Fig. 3b,c). Differences in protein degradation rates are therefore unlikely to account for the accumulation of BCL-XL and BCL-W in DIS cells.

We next examined if the translation rates of BCL-XL change in DIS cells. We separated ribosomes of control and senescent cells by sucrose gradient fractionation into monosomes (fraction #1, untranslated) and polyribosomes (polysomes, fractions #2−5) (Fig. 3f). In DIS cells we found a lower polysomal content and a correspondingly higher monosomal content than in the control cells (23 versus 37%, respectively, for polysomes and 77% versus 63%, respectively, for monosomes; Fig. 3f). Thus, as reported for other types of cellular stresses^29^, global translation in DIS cells is reduced. Next, we calculated the distribution of mRNA content in the different ribosomal fractions. The distribution of GAPDH and actin mRNAs in these fractions remained unchanged between control and senescent cells. Thus, these genes can represent controls whose translation levels in the two cell types tested are similar (Fig. 3g). We then examined the polysomal distribution of p53 mRNA, whose protein level in senescent cells is upregulated (Fig. 1d). Because the expression of p53 is regulated mainly by the proteasome (Supplementary Fig. 3b), there was no change in the distribution of its mRNA between the different ribosomal fractions (Fig. 3g). Unlike p53, however, the distribution of *BCL-XL* mRNAs between the ribosomal fractions showed a significant shift to heavier polysomal fractions. In DIS cells, we detected a significant increase in the presence of BCL-XL in fraction #3 relative to growing controls (Fig. 3g). This effect was accompanied by a tendency towards reduced presence of *BCL-XL* in the untranslated (fraction #1) and relative light-polysomal fraction (fraction #2). These results are consistent with increased translation rates of BCL-XL in DIS cells.

Cellular stress influences protein translation in mammalian cells^30^. The mechanisms regulating BCL-XL translation in senescent cells following DNA damage may rely on both cap-dependent and cap-independent translation, as previously shown for BCL-2 (ref. 31). The mechanistic target of rapamycin (mTOR) regulates cap-dependent translation by controlling the phosphorylation and activity level of translation initiation factor 4E-binding proteins (4EBPs) and S6 kinases (S6K1 and S6K2) (ref. 32). We found reduced mTOR activity in DIS cells relative to control (Supplementary Fig. 3d) consistent with its known inhibition by p53. Further inhibition of mTOR in these cells by rapamycin did not affect BCL-W or BCL-XL protein levels (Supplementary Fig. 3e).

This suggested that cap-independent mechanisms regulate BCL-W and BCL-XL protein translation in DIS cells. The 5′-untranslated region (UTR) of the *BCL-XL* transcript contains an internal ribosome entry site (IRES) element that may mediate the regulation of its translation by cap-independent initiation^33^. To test if BCL-XL translation is governed by its IRES motif, growing control (G) and DIS cells were transduced with bicistronic constructs containing the *BCL-XL* IRES positioned between two luciferase cistrons, such that the IRES was upstream to firefly luciferase gene (Fig. 3h). Cells were subjected to a dual-luciferase reporter assay and BCL-XL IRES activity was calculated by measuring the Firefly to Renilla (Fluc/Rluc) ratio of luciferase luminescence. As cap-dependent translation is inhibited in DIS cells (Supplementary Fig. 3d), we used a control vector that lacks an IRES element as reference^34^. A significant increase in *BCL-XL* IRES activity was detected in DIS cells relative to growing controls (G) (Fig. 3i). Increased BCL-XL translation in DIS cells might therefore be governed by its IRES motif. Altogether, our findings reveal that the increased expression of BCL-XL and BCL-W in senescent cells is regulated by a combination of transcriptional and translational mechanisms.

### ABT-737 eliminates senescent cells after DNA damage *in vivo*

**Figure 4:**
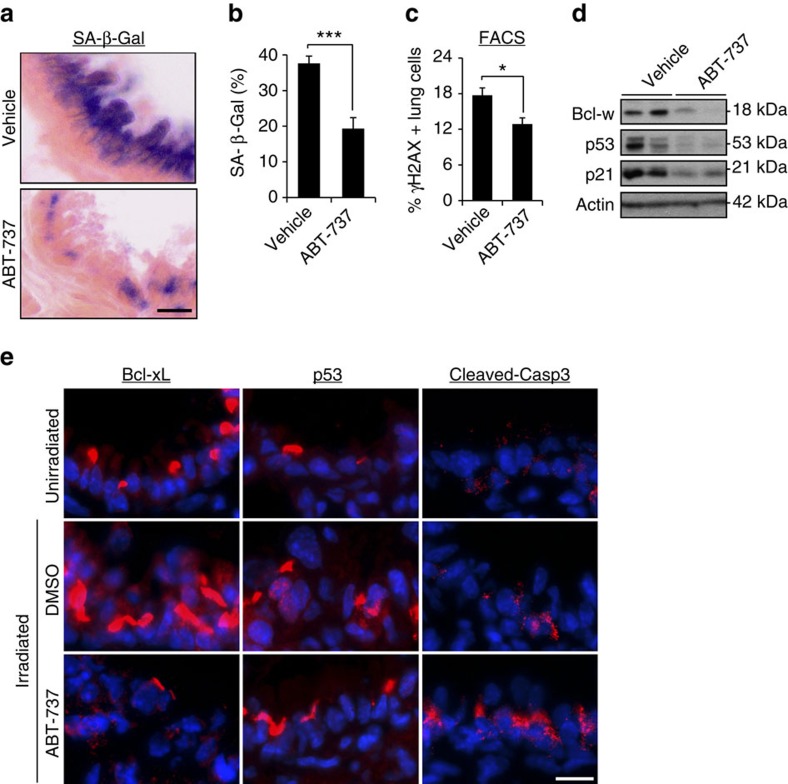
ABT-737 eliminates senescent cells from lung of irradiated mice. (**a**) Seven days post irradiation, mice were treated with ABT-737 (*n*=5) or vehicle (*n*=5) for 2 consecutive days. Lungs were dissected 1 day thereafter, sectioned and the sections were stained for SA-β-Gal. Scale bar, 10 μm. (**b**) Quantification of SA-β-Gal staining in the bronchioles area of vehicle- or ABT-737-treated mice. Data were presented as means±s.e.m. from five mice in each group. (**c**) One day following vehicle or ABT-737 treatment, lung were dissociated, stained for γH2AX and analysed by FACS. Percentage of γH2AX-positive cells was quantified from this analysis. Data were presented as means±s.e.m. from five mice in each group. (**d**) Western blot analysis of Bcl-w, p53 and p21 in the mice described in **a**. Two mice represent each condition, each line represents a mouse. (**e**) Lung sections from unirradiated mice and the mice described in **a** were stained for Bcl-xL, p53 and cleaved caspase-3, a marker of apoptosis. Scale bar, 10 μm. Data in **b**,**c** were analysed using Student's *t*-test. *P<0.05, ***P<0.0005.

Our experiments above showed that ABT-737 treatment causes selective elimination of senescent cells in tissue culture, including of cells that were induced to senesce by direct induction of DNA damage. We therefore set out to test the effectiveness of ABT-737 treatment in elimination of DNA-damage-induced senescent cells *in vivo*. To this end, we induced lung damage and senescence in mice by ionizing radiation, which causes long-lasting accumulation of senescent cells, readily identified by persistent DNA damage, in the lungs^35^. Seven days after irradiation the mice were treated with ABT-737 for 2 days, and 1 day later the lungs were excised and analysed for the expression of senescence markers. SA-β-Gal staining showed a significant decrease in the amount of senescent cells following ABT-737 treatment (Fig. 4a,b). This reduction was accompanied by a significant decrease in the numbers of γH2AX-positive lung cells (Fig. 4c) and a decrease in the expression of the senescence markers p53 and p21 (Fig. 4d,e). The molecular targets of ABT-737, BCL-XL and BCL-W, were expressed in the irradiated lungs and their levels were reduced as a result of the treatment (Fig. 4d,e). The reduction in the expression of senescence markers and ABT-737 target proteins was accompanied by increased caspase-3 cleavage, suggesting an increase in apoptosis in the lung following the treatment. These findings establish that ABT-737 treatment leads to the elimination of DIS cells *in vivo*.

### ABT-737 eliminates p53-induced senescent cells from skin

**Figure 5:**
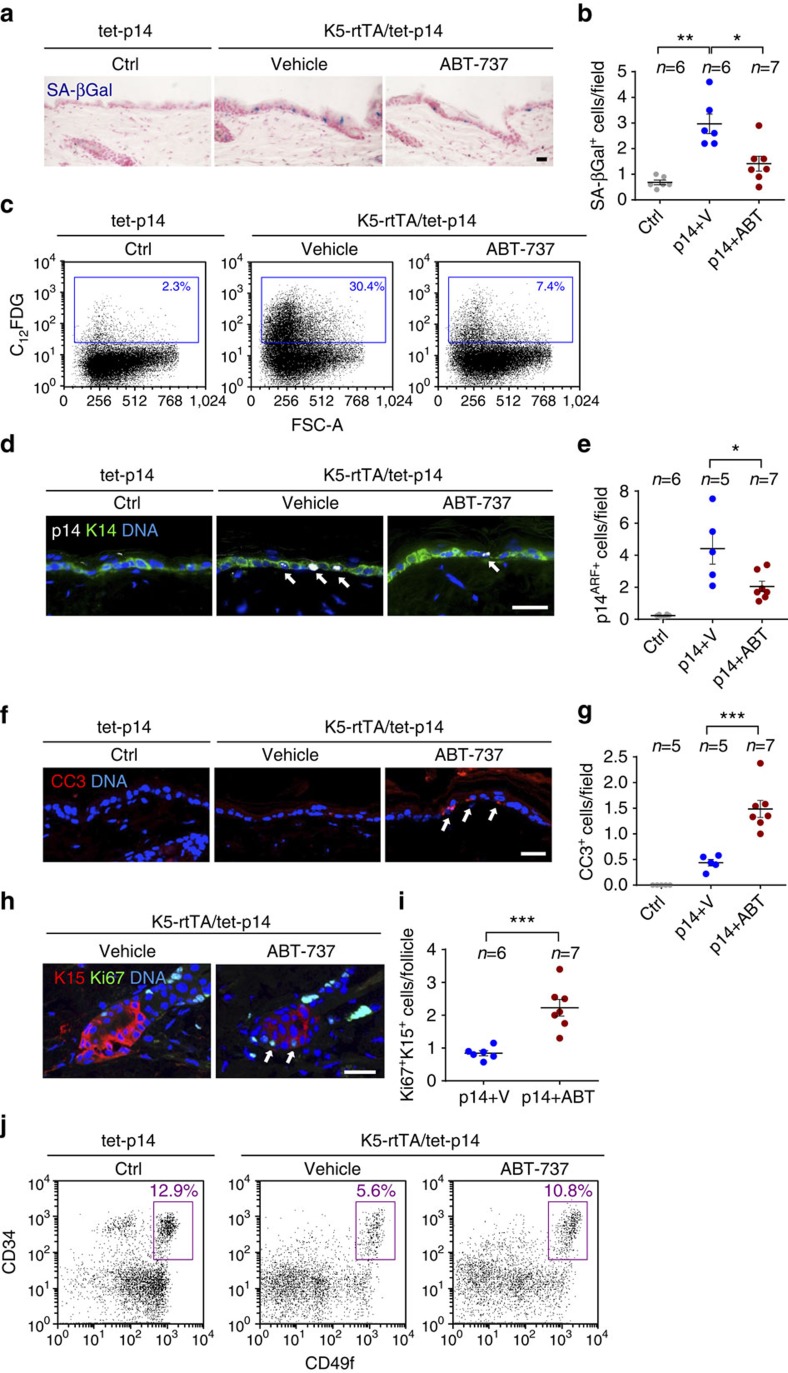
ABT-737 treatment eliminates senescent epidermal cells and induces stem cell proliferation. (**a**) Skin sections stained for SA-β-Gal (blue) of control tet-p14 (Ctrl) mice, and K5-rtTA/tet-p14 mice treated with dox for 4 weeks to activate p14^ARF^, and subsequently treated with ABT-737 (p14+ABT) or vehicle (p14+V) for 4 consecutive days. (**b**) SA-β-Gal^+^ cells per microscopic field in mice as in **a**. (**c**) FACS analyses of SA-β-Gal activity (C_12_FDG stain) in epidermal cells isolated from indicated mice. Gate indicates SA-β-Gal^+^ cell percentages. FSC-A—forward scatter. (**d**) Skin sections stained for the human p14^ARF^ (white, arrows) from indicated mice after 2 days of ABT-737 or vehicle treatment. K14 (green) marks the basal epidermis. (**e**) p14^ARF+^ cells per field in mice as in **c**. (**f**) Sections of mice as in **c** stained for the apoptosis marker cleaved caspase-3 (CC3, arrows). (**g**) CC3^+^ cells per field in same mice. (**h**) Sections of hair follicle bulges of p14-expressing mice after 4 days of ABT-737 or vehicle treatment, stained for the proliferation marker Ki67 (green, arrows) and the bulge marker K15 (red). (**i**) Numbers of Ki67^+^K15^+^ cells per follicle per mouse in mice as in **h**. Dots represent mean number in individual mice, combining three independent experiments. (**j**) Representative FACS analyses of epidermal cells from indicated mice after 2 days of ABT-737 or vehicle treatment, stained for CD34, CD49f and Sca1. Charts show only Sca1^–^ (follicular) cells. Gate indicates percentage of CD34^+/^CD49f^high^ hair-follicle stem cells. *n*=2 mice per group, experiment was done twice. Throughout, dots indicate individual mice, bars indicate mean±s.e.m. Data in **b**,**e**,**f** show mice combined from two independent experiments out of four conducted. *P<0.05; **P<0.005; ***P<0.0005 by Student's *t*-test. Scale bars, 25 μm.

We next tested whether BCL protein family inhibition by ABT-737 could eliminate senescent cells induced by direct activation of p53 in the skin. To this end, we used double-transgenic K5-rtTA/tet-p14 mice, in which the human *p14*^*ARF*^ gene is inducibly expressed in the basal layer of the skin epidermis^36^. Induction of *p14*^*ARF*^ in these mice activates p53 and generates senescent epidermal cells that are retained in the tissue for weeks^36^. To generate senescent cells, we activated expression of *p14*^*ARF*^ in 3-week-old mice for a period of 4 weeks, and then treated the mice with ABT-737 for 4 consecutive days. The number of senescent cells in the epidermis, determined by SA-β-Gal staining, was dramatically reduced in the ABT-737-treated mice relative to vehicle-treated mice (Fig. 5a–c). A similar degree of elimination was observed after ABT-737 treatment of these mice for 2 days (data not shown). Concomitantly, the percentage of epidermal cells in which the transgenic p14^ARF^ protein could be detected was reduced (Fig. 5d,e), indicating preferred elimination of transgene-expressing cells. Increased levels of apoptosis were detected in the epidermis after 2 days of ABT-737 treatment (Fig. 5f,g), consistent with increased apoptosis as the mechanism of senescent cell elimination. These findings indicate that the survival signal provided by BCL-family proteins is an essential component of the ability of senescent cells to be retained in the tissue, and in its absence they rapidly die.

Upon p14^ARF^ activation in mice at 3 weeks of age nearly all hair follicles are arrested in the resting (telogen) state, and only rare stem cells in the bulge express the proliferation marker Ki67 (ref. 36) (Fig. 5h,i). Interestingly, we found that treatment with ABT-737 after 2–4 weeks of p14^ARF^ induction led to an increase in the numbers of proliferating hair-follicle stem cells in the bulge (Fig. 5h,i). This effect was also evident three days after treatment was stopped (Supplementary Fig. 4). Furthermore, the number of CD34^+^/CD49f^high^ bulge stem cells was increased following treatment (Fig. 5j). These findings suggest that the elimination of senescent hair-follicle stem cells allows other (non-senescent) bulge cells to initiate proliferation and repopulate the stem cell compartment.

## Discussion

Our findings reveal that BCL protein family activation is a central molecular mechanism by which senescent cells acquire increased resistance to apoptosis, and which supports their retention within tissues. The resistance of senescent cells to apoptosis in response to acute damage might be beneficial for the organism, as these cells may assist in the maintenance of tissue integrity during repair; once their presence is no longer required, senescent cells may be eliminated by the immune system^5^,^15^. This resistance may also help to sustain senescent cells during embryonic development^37^,^38^,^39^. In cases of chronic wounding, however, senescent cells may accumulate owing to variation in immune surveillance between different tissues and in different pathological conditions. Such accumulation can become deleterious to the organism^3^.

We found that BCL-XL and BCL-W are central among BCL-family members in supporting DIS cell survival, and these proteins are upregulated through a combination of increased transcription and cap-independent translation. Regulation of BCL-W expression in all of the types of senescent cells we tested is mainly achieved by transcriptional regulation of the short variant of the gene, which is transcribed from an alternative transcription start site. Because of the increase in the mRNA levels of BCL-W, the identification of differences in translation was not possible by the polysome separation method we employed. Therefore, we cannot exclude the possibility that translational regulation also plays a role in the expression of BCL-W, similarly to the increased specific translation we detected for BCL-XL in DIS cells. This specific translation is driven by the IRES element present in the 5′-UTR of the gene, which has a proven ability of enhancing cap-independent translation^33^. Of note, cap-dependent translation is inhibited in response to various forms of cellular stress^29^. Cellular stress induced by the DNA damage response is a central mechanism that drives senescence in aging and age-related diseases^2^,^3^,^12^. Therefore, in senescent cells accumulating in these conditions the activation of specific translation modes might be the central mechanism that leads to BCL-XL upregulation and resistance to apoptosis. In contrast to these cells, oncogene-induced senescent cells have increased mTOR signalling leading to an increase in global translation^40^,^41^. In this situation, the observed increase in mRNA levels can lead to accumulation of BCL-W and BCL-XL in the cells and support their viability. It is possible, therefore, that the regulation of the expression of BCL-XL and BCL-W proteins is dependent on the specific context of senescence induction.

Importantly, we show here that inhibition of BCL-W and BCL-XL specifically induces apoptosis of senescent cells both *in vitro* and *in vivo*. This represents one of the first demonstrations of efficient targeted pharmacological removal of senescent cells from a living tissue—senolytic drug treatment. We show the senolytic activity of ABT-737 in two independent models of senescence *in vivo*: DIS in the lungs, caused by whole-body irradiation, and a transgenic model of senescence in the skin, induced by p53 activation in the absence of DNA damage.

In the lung, large numbers of senescent cells expressing Bcl-xl, and the DNA damage marker γH2AX, were detected following irradiation, and these cells were eliminated by the treatment. In the skin, we have previously shown that p14^ARF^ expression leads to the accumulation of senescent cells in the epidermis, and that these cells are retained for weeks^36^. Here we show that treatment with ABT-737 eliminates most of these senescent cells, indicating that an anti-apoptotic signal is central to their retention in the tissue. In addition, p14^ARF^ activation causes hair-follicle stem cells to arrest irreversibly, preventing follicle growth^36^. We show here that ABT-737 allows re-entry of some of these bulge stem cells into the cell cycle, leading to an increase in overall stem cell numbers. The relevance of these observations to different tissues and conditions is strongly supported by similar recent observations in the haematopoietic system^42^. We did not observe, however, prominent regrowth of hair in these mice during the time frame of our experiment, suggesting that the overall numbers of activated stem cells accumulated during this time are not sufficient to execute this task, or that additional damage to the hair follicle prevents their regrowth. Overall, these findings suggest that the clearance of senescent cells can lead to tissue renewal and improved fitness, a possibility consistent with the recent finding that elimination of senescent cells via transgenic techniques and combinatorial treatments improves aging-associated phenotypes^14^,^43^.

The ability to pharmacologically eliminate senescent cells *in vivo* opens the door to study the roles of senescent cells in a wide range of physiological settings in which they are detected, and to dissect their beneficial and detrimental functions. Importantly, this is an early step towards potential clinical application of senolytic drugs, in such settings as aging-associated diseases. The chemotherapeutic elimination of senescent cells from premalignant lesions and tumours may also prove beneficial, in particular settings in which a pro-tumorigenic function of senescent cells in the tumour or stroma will be proven. Overall, our findings reveal a central molecular mechanism maintaining the viability and retention of senescent cells in tissues, and suggest that the elimination of senescent cells by inhibition of this mechanism represents a promising strategy for targeting senescent cells during tumourigenesis and age-related diseases.

## Experimental procedures

### Cell culture

Human IMR-90 fibroblasts were obtained from ATCC. MEFs were isolated according to standard procedures^44^. Cells were maintained in DMEM supplemented with 2 mM L-glutamine, 100 units per ml of penicillin, 100 μg ml^−1^ of streptomycin and 10% fetal bovine serum. DNA DIS was introduced by etoposide treatment (E1383, Sigma) at a concentration of 50 μM for 48 h. Cells acquired the senescence phenotype 7 days post treatment. OIS was introduced using retroviral infection with mutated H-Ras (pLPC mCherry-H-Ras12V plasmid^45^) at 3% oxygen. Empty vector-transduced cells served as controls. Cells acquired the senescence phenotype 7 days post selection. RS was induced by long-term passaging of the cells in tissue culture. Cells acquired the senescence phenotype after 40 population doublings.

### Apoptosis assay

Target cells were plated in 12-well plates at 1.2 × 10^5^ cells per well. Twenty-four hours after seeding, cells were treated with 75 ng ml^−1^ TNF-α (210-TA-020, R&D Systems) and 10 μg ml^−1^ CHX (C1988, Sigma) for 10 h. The percentage of survival was determined based on quantification of remaining adherent cells using PrestoBlue reagent (A13262, Life Technologies Ltd.) relative to control DMSO treated cells.

### UV sensitivity assay

Target cells were plated as described above. Twenty-four hours after seeding cells were washed and incubated with Hank's buffer and irradiated with UV-C at 254 nm using a low-pressure mercury lamp (TUV 15w G15T8, Philips). UV dose rate was measured using an UVX Radiometer (UVP) equipped with a 254 nm detector. After irradiation, the cells were incubated in fresh medium for an additional 24 h. Remaining adherent cells were quantified using PrestoBlue reagent relative to non-irradiated cells.

### Cell survival assays

Target cells were plated as described above, and the indicated molecules were added to each well at the indicated concentrations for 24 h. ABT-737 and Obatoclax were purchased from Selleck Chemicals (S1002 and S1057, respectively). ABT-199 was purchased from ChemieTek (CT-A199). Z-VAD-FMK was purchased from Santa Cruz Biotechnology (SC-311561A) and was added to the cells 4 h before ABT-737 addition at a concentration of 100 μM.

### Immunoblotting

Cell lysates (15-30 μg of protein) were resolved by 15% SDS–polyacrylamide gel and transferred onto Immobilon P membrane (IPVH00010, Millipore). After blocking with 5% BSA in TBST (TBS with 0.01% Tween 20) for 1 h, the membranes were probed with antibodies against cleaved-PARP (#9541), cleaved-ICAD (#9731), cleaved-caspase-3 (#9661), BCL-W (#2724), BCL-XL (#2764) and Bcl-2 (#2870) (all from Cell Signaling), Mcl-1 (1239-1, Epitomics), p16 (sc-1207, Santa Cruz) and β-Tubulin (sc-9104, Santa Cruz), p21 (556431, BD Bioscience), p53 (mix of DO-1 and PAb1801 kindly provided by Prof. Moshe Oren, Weizmann Institute of Science) and β-Actin (A5441, Sigma). Antibodies were visualized with chemiluminescence detection (34080, Thermo Fisher Scientific, USA). For proteasomal degradation analysis, cells were incubated with MG-132 (BML-PI102-0005, Enzo) in the indicated concentrations and times. The scans of the most important blots are shown at Supplementary Fig. 5.

### siRNA

Cells were transfected with 50 nM of ON-TARGETplus SMARTpool siRNA targeting Bcl-2, BCL-W, BCL-XL (L-003307-00-0005, L-004384-00-0005 and L-0030458-00-0005, respectively) or with Non-targeting siRNA pool (D-001810-10-20) as control (all from Thermo Scientific). Transfections were performed overnight. Four days post transfection remaining adherent cells were quantified using PrestoBlue reagent.

### Quantitative real-time PCR

Total RNA was extracted using RNeasy kit (74104, Qiagen), followed by DNase I treatment. cDNA was produced using random hexamers. The cDNA samples were amplified using Platinum SYBR Green qPCR SuperMix (11744-500, Life Technologies) in StepOnePlus Real-Time PCR System (Applied Biosystems). The relative expression was normalized using the expression levels of GAPDH or Actin. Primer sequences are found in Supplementary Table 1.

### Polysome profiling

Cells were seeded in 15 cm plates. Forty-eight hours later cells were incubated for 5 min with 100 μg ml^−1^ CHX, washed with PBS+CHX, harvested and resuspended in 0.5 ml of modified LBA buffer (20 mM Tris, pH 8, 140 mM KCl, 5 mM MgCl, 100 μg ml^−1^ CHX, 1.8 mM DTT, 200 U ml^−1^ Rnasine, protease inhibitor cocktail, phosphatase inhibitor cocktail, both from Sigma)^34^. Next, Triton X-100 and deoxycholate were added to a final concentration of 0.5% each for lysis of 5 min on ice followed by 5 min centrifugation at 12,000*g*. Equal OD units (260 nm) from each sample were loaded onto 10–50% linear sucrose gradients and centrifuged at 260,173*g* (39,000 r.p.m.) for 100 min at 4 °C. Gradients were fractionated from the top into 200 μl fractions (51 in total) and RNA was measured manually by NanoDrop (NanoDrop 2000c, Thermo). After profiling, the OD fractions were merged and RNA was extracted from each fraction using TRI-Reagent (Sigma) and Direct-zol RNA MiniPrep kit (R2050, Zymo Research) according to the manufacturer's protocol. RNA was resuspended in 25 μl RNase-free water. For PCR with reverse transcription reaction, 1 μg of RNA was taken from the different fractions of each gradient, following by PCR with reverse transcription.

### Dual-luciferase assay

The bicistronic construct of BCL-XL 5′-UTR and the control construct were kindly provided by A. Kimchi (Weizmann Institute). For dual-luciferase assays, 7 × 10^4^ IMR-90 cells were plated per well in 24-well plates, 24 h before transfection. Cells were transfected with the bicistronic construct using TurboFect Transfection Reagents (Thermo Fisher Scientific). Renilla and Firefly luciferase activities were measured 48 h post transfection, using the Dual-Luciferase Reporter Assay System (Promega, USA), according to manufacturer's protocol, with a Veritas Luminometer (Promega, USA). Relative IRES activity was determined by calculating the Firefly luciferase /Renilla luciferase luminescence ratio.

### Mice

All experiments were done with approval from the Weizmann Institute and the Hebrew University Animal Care and Use Committees. K5-rtTA/tet-p14 transgenic mice and single transgene sibling controls, (C57Bl/129sv background) received doxycycline (2 mg ml^−1^, Sigma) in their drinking water at 3 weeks of age for activation of the p14^ARF^ transgene. After 2–4 weeks of transgene activation, ABT-737 (75 mg kg^−1^ in 30% propylene glycol, 5% Tween 80, 3.3% dextrose in water pH 4–5), or vehicle, was injected into p14^ARF^-expressing mice intraperitoneally for 2 or 4 consecutive days. Mice were then shaved and sacrificed, and back skins were paraffin embedded for immunohistology, or frozen in OCT solution for cryosectioning and SA-β-gal stains. For induction of DNA damage and senescence, male mice at the age of 6–8 weeks were irradiated with 8 Gy. Seven days post irradiation, mice were injected i.p. with 75 mg kg^−1^ ABT-737 or vehicle for 2 days. Mice were killed 24 h after the last ABT-737 injection.

### Immunohistology

Immunohistology was performed according to standard procedures on 5 μm paraffin sections, using Peroxidase Substrate kits (Vector) or fluorescently labelled secondary antibodies (Jackson). Antibodies used: p14^ARF^ (Abcam, ab3642), CC3 (Cell Signaling, #9661), Ki67 (Labvision, RM-9106), K14 (Progen, GP-CK14) and K15 (Santa Cruz sc-56520). For SA-β-Gal stains, 10-12 μm cryosections of OCT-embedded mouse skins were fixed in 0.5% glutaraldehyde for 15 min and stained as previously described^36^. Bright field images were collected using an Olympus CX41 and DS-Fi1 camera and processed using NIS Elements software (Nikon). Fluorescent images were collected using an Olympus FV1000 confocal microscope. SA-β-Gal staining in bronchioles area was quantified using ImageJ, and by visual scoring in the skin. Greater than 10 microscopic fields were scored in each sample in all stains. Proliferating hair-follicle stem cells were scored by counting the number of K15^+^Ki67^+^ cells per follicle, in >15 fields in each mouse (1–3 follicles per field); values indicate mean numbers per individual mice.

### FACS analysis

Adult epidermal cells were isolated using the procedure described in ref. 46 with minor modifications. Mouse back skins were shaved and excised. The dermal side of the skin was scraped with a scalpel in to remove adipose and muscle tissue. The skin was floated in trypsin 0.25% EDTA solution overnight at 4 °C, and the epidermis was then scraped off, minced and suspended in a trypsin-PBS 0.5% BSA solution, and filtered through a 70 μm cell strainer followed by filtration through a 40 μm cell strainer. A total of 1 × 10^6^ live epidermal cells were resuspended in 100 μl PBS containing 0.5% BSA and incubated for 30 min at 4 °C with the following conjugated antibodies: CD34 (BD Bioscience 562608), CD49f (eBioscience 17-0495-80) and Sca1 (Biolegend 108113). SA-β-Gal activity was assayed using the fluorescent β-galactosidase substrate C_12_FDG (Invitrogen D-2893), as described^47^. Samples were analysed using a MACSQuant fluorescence-activated cell sorting (FACS) analyser (Miltenyi). C_12_FDG (33 μM) was added to 1 × 10^6^ live suspended cells and incubated for 1 h at 37 °C with gentle shaking, and fluorescent cells were scored by FACS. For γ-H2AX quantification in lung cells, lungs were minced and dissociated to a single-cell suspension by incubation for 1 h with RPMI supplemented with 0.5 mg ml^−1^ Collagenase IV and 0.02 mg ml^−1^ DNase I at 37 °C. Cells were then filtered with 100 μM nylon filter mesh, washed twice with PBS and incubated overnight with anti-γ-H2AX primary antibodies (Cell Signaling). Cells were washed twice and incubated for 45 min with secondary antibodies (Jackson ImmunoResearch), followed by two additional washing and 10 min staining with DAPI. Cells were then analysed by FACS.

### Statistical analysis

The data were presented as mean±s.e.m. Statistical significance was determined using Student's *t*-test. The criterion for statistical significance was considered *P*<0.05.

## Additional information

**How to cite this article:** Yosef, R. *et al*. Directed elimination of senescent cells by inhibition of BCL-W and BCL-XL. *Nat. Commun.* 7:11190 doi: 10.1038/ncomms11190 (2016).

## Supplementary Material

Supplementary Information Supplementary Figures 1-5 and Supplementary Table 1
