# Supplementary material for: Directed elimination of senescent cells by inhibition of BCL-W and BCL-XL
Source: Nat Commun. 2016 Apr 6;7:11190. doi: 10.1038/ncomms11190 (PMC4823827; doi:10.1038/ncomms11190)
Supplement: Supplementary Information — Supplementary Figures 1-5 and Supplementary Table 1 [file ncomms11190-s1.pdf]

Supplementary figures

Directed Elimination of Senescent Cells by Inhibition of BCL-W and BCL-XL

Reut Yosef, Noam Pilpel, Ronit Tokarsky-Amiel, Anat Biran, Yossi Ovadya, Snir Cohen, Ezra Vadai, Liat Dassa, Elisheva Cohen, Reba Condiotti, Ittai Ben-Porath, Valery Krizhanovsky

Yosef_Supplementary Figure 1.

a

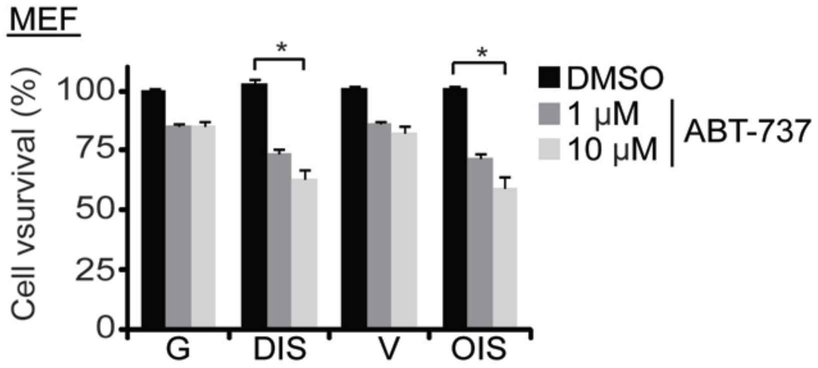

Supplementary figure 1: ABT-737 eliminates senescent MEFs. (**a**), Percent survival of MEF cells, either proliferating (G – vehicle treated, V – empty vector transfected), or DNA-damage induced senescent (DIS) and oncogene induced senescent (OIS), following 24hr treatment with the indicated concentrations of ABT-737. The treatment induced preferential decrease in the viability of senescent cells. Data are presented as mean±s.e.m of three repeats, performed in triplicates. Data was analyzed using Student's t-test. *$P < 0.05$.

Yosef_Supplementary Figure 2.

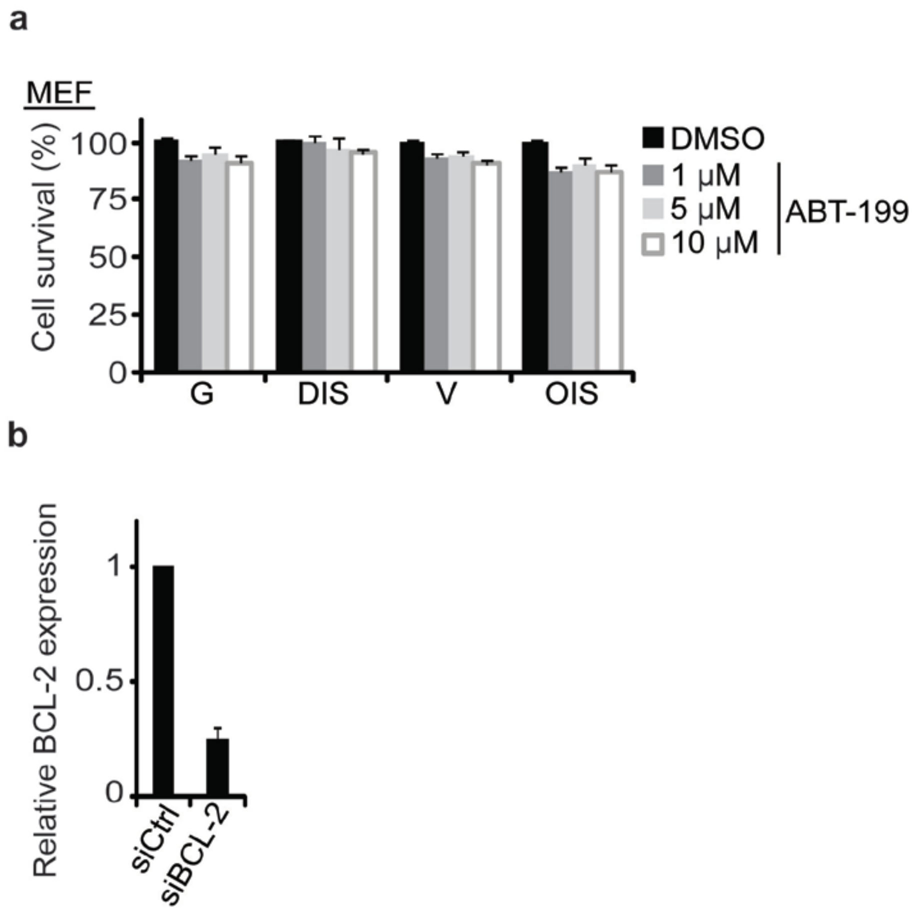

Supplementary figure 2: BCL-2 inhibition had no effect on the viability of MEFs. (**a**) Percent Survival of DNA-damage induced senescent (DIS) and oncogene induced senescent (OIS) MEFs and growing (G) or vector transduced (V) control cells following treatment with the indicated concentrations of ABT-199 for 24 hr. Data are presented as mean±s.e.m of three repeats, performed in triplicates. (**b**) mRNA expression level of BCL-2 in DNA-damage induced senescent (DIS) IMR-90 cells following transfection with siRNA against BCL-2 or control siRNA. 75% reduction in mRNA level of BCL-2 following siRNA transfection was observed. mRNA means with s.e.m. of triplicates are displayed.

Yosef_Supplementary Figure 3.

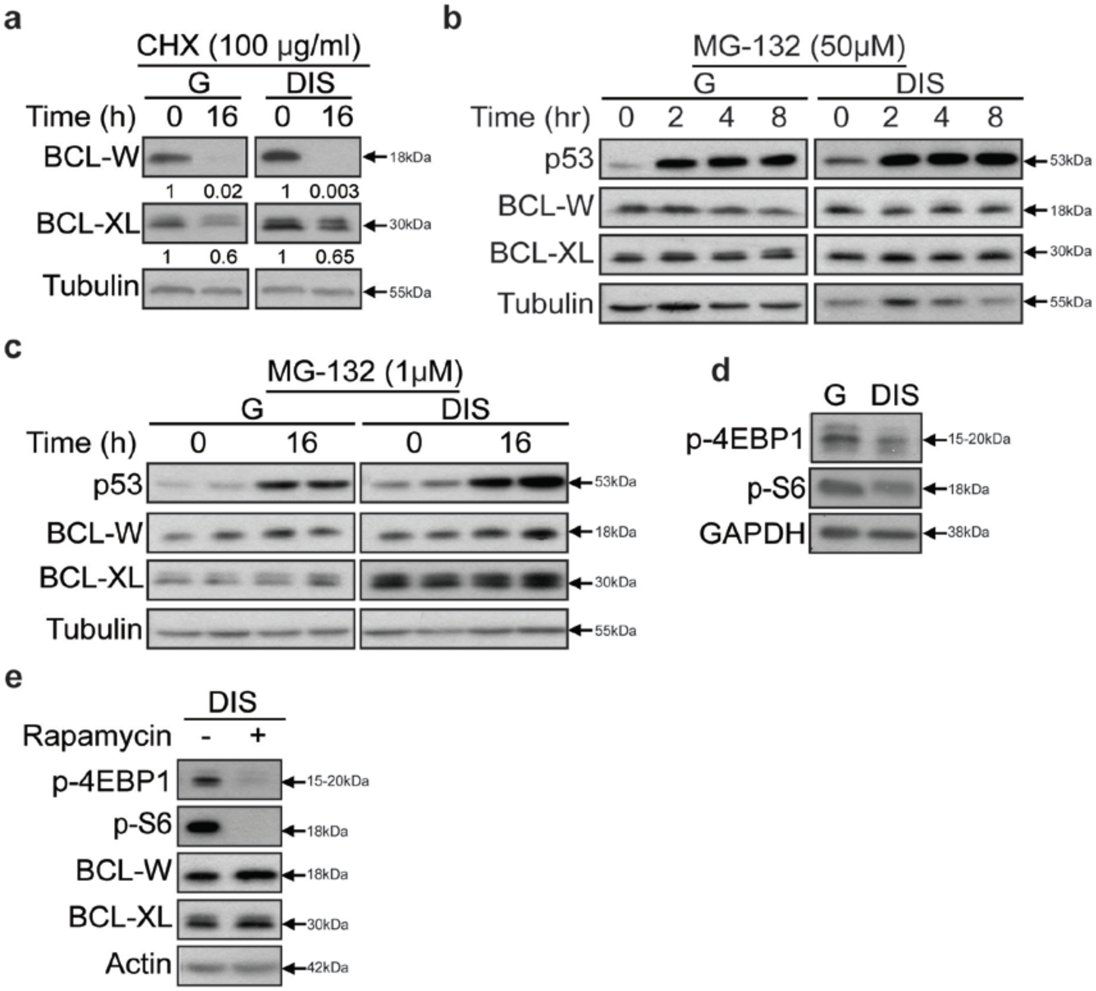

Supplementary figure 3: Posttranscriptional regulation of BCL-W and BCL-XL in senescent cells. (**a**) Western blot analysis of BCL-W and BCL-XL in DIS and control (G) cells before and after 16 hours cycloheximide treatment (CHX). Translation inhibition by CHX for long period of time (16hr) didn't show any change in protein stability between growing and DIS cells; representative blot of three independent experiments. (**b-c**) Western blot analysis of BCL-W, BCL-XL and p53 in DIS and control (G) cells following treatment with the proteasome inhibitor MG-132 for time periods indicated. Proteasome inhibition for short or long periods of times (2-8hr or 16hr, respectively), at low or high concentrations

(1µM or 50µM, respectively) didn't yield any change in protein level of BCL-XL and BCL-W; representative blot of three independent experiments. (**d**) Western blot analysis of phosphorylated 4EBP1 and S6 (p4EBP1 and pS6) in G and DIS cells; representative blot of three independent experiments. (**e**) Western blot analysis of BCL-W, BCL-XL and p4EBP1 and pS6 in DIS cells following 72 hours treatment with 0.1µM of mTOR inhibitor Rapamycin or vehicle. Representative blot of three independent experiments are shown.

Yosef_ Supplementary figure 4

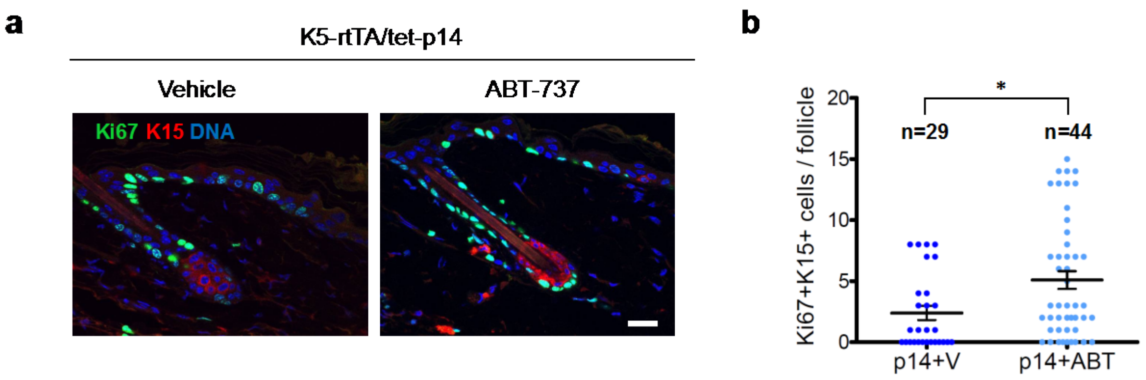

Supplementary Figure 4. Increased hair follicle stem cell proliferation 3 days subsequent to ABT-737 treatment. (a) Sections of hair follicle bulges stained for the bulge marker K15 (red) and the proliferation marker Ki67 (green) from $p14^{ARF}$-expressing mice treated with ABT-737 for 2 days, and sacrificed 3 days subsequent to the last treatment. (b) Number of Ki67-positive cells in individual bulges from ABT-737 treated (*n* = 3) and vehicle treated (*n* = 2) mice. Dots indicate individual follicles from scored mice, bars indicate mean ± s.e.m. * $P < 0.05$ by Student's *t*-test.

Yosef_Supplementary Figure 5.

Figure 1B.

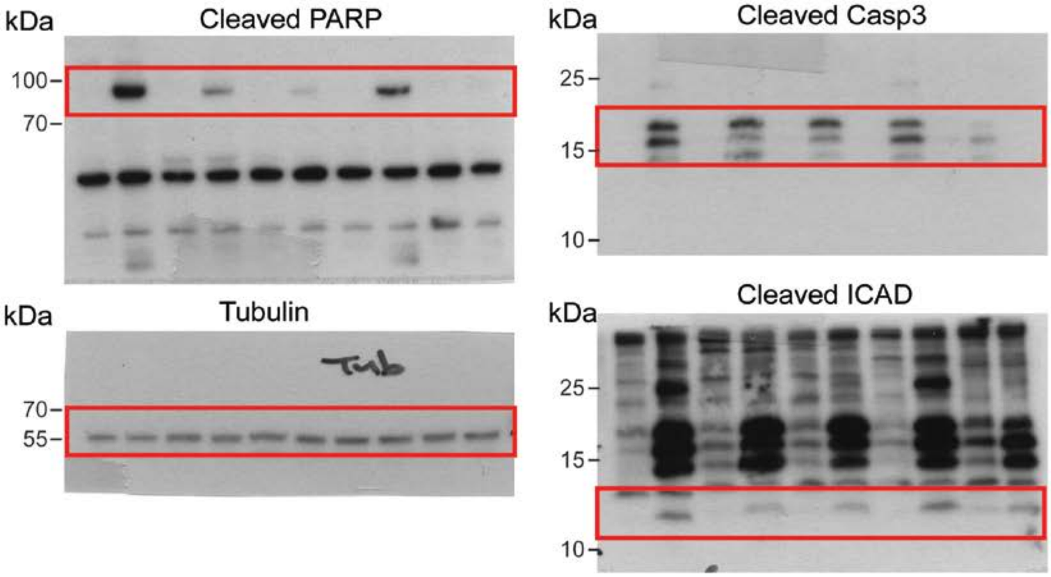

Figure 1G.

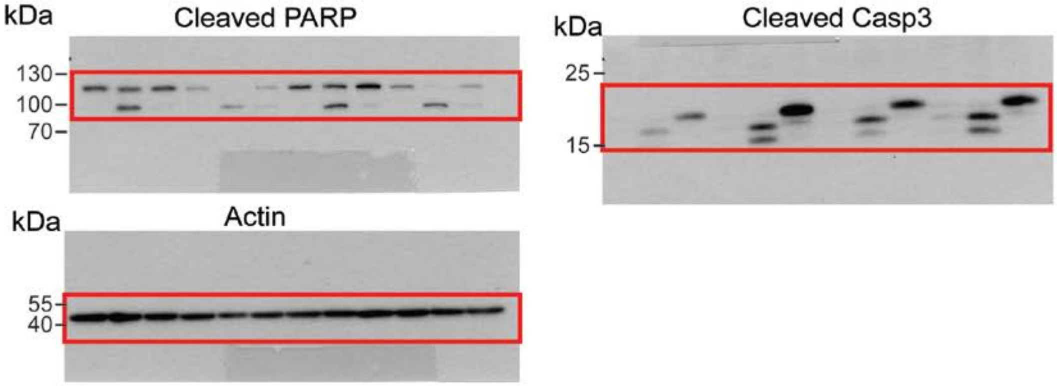

Figure 2D.

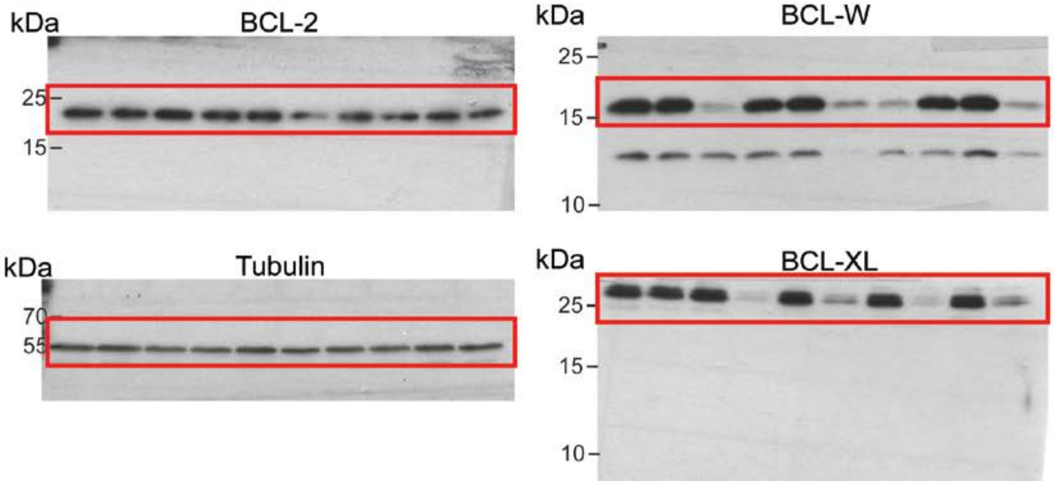

Supplementary Figure 5. Uncropped images of western blots for figures 1B, 1G, 2D. Red boxes indicate the part of the blot used for cropped presentation.

Supplementary tables

Supplementary Table 1: Quantitative RT-PCR primers.

BCL-2<br>(NM_000633)	5'- AACATCGCCCTGTGGATGAC -3'<br>5'- GGCCGTACAGTTCCACAAAG - 3'
BCL-W v1<br>(NM_004050)	5'- AGCTCCTGCACCAGGAAAC -3'<br>5'- GCCAGCTCCACAGACATAAC -3'
BCL-W v2<br>(NM_001199839)	5'- GACCCGTGAGATCCCTAACCT -3'<br>5'- TGGGGCCTTTCATCCTCCT -3'
BCL-XL<br>(NM_138578)	5'- GGCCACTTACCTGAATGACC -3'<br>5'- AAGAGTGAGCCCAGCAGAAC -3'
p21<br>(NM_000389)	5'- TGTCTTTCCTGGCACTAACG -3'<br>5'- AAACAGTCCAGGCCAGTATG -3'
Actin<br>(NM_001101)	5'- TTCAACACCCCAGCCATGT -3'<br>5'- GCCAGTGGTACGGCCAGA -3'
GAPDH<br>(NM_002046)	5'- GACAGTCAGCCGCATCTTC -3'<br>5'- CGTTGACTCCGACCTTCAC -3'
